# The economic burden of prostate cancer – a Swedish prevalence-based register study

**DOI:** 10.1186/s12913-020-05265-8

**Published:** 2020-05-20

**Authors:** Shuang Hao, Ellinor Östensson, Martin Eklund, Henrik Grönberg, Tobias Nordström, Emelie Heintz, Mark Clements

**Affiliations:** 1 Department of Medical Epidemiology and Biostatistics, Karolinska Institutet, Nobels väg 12A, 171 65 Stockholm, Sweden; 2 Department of Women’s and Children’s Health, Karolinska Institutet, Tomtebodavägen 18A, 171 77 Stockholm, Sweden; 3 Department of Clinical Sciences, Danderyd Hospital, Mörbygårdsvägen, 182 88 Danderyd, Sweden; 4 Department of Learning, Informatics, Management and Ethics, Karolinska Institutet, Tomtebodavägen 18A, 171 77 Stockholm, Sweden

**Keywords:** Costs-of-illness, Prostate cancer, Prostate-specific antigen test, Sweden

## Abstract

**Background:**

Incidence and prevalence of prostate cancer in Sweden have increased markedly due to prostate-specific antigen (PSA) testing. Moreover, new diagnostic tests and treatment technologies are expected to further increase the overall costs. Our aims were (i) to estimate the societal costs for existing testing, diagnosis, management and treatment of prostate cancer, and (ii) to provide reference values for future cost-effectiveness analyses of prostate cancer screening and treatment.

**Methods:**

Taking a societal perspective, this study aimed to investigate the annual cost of prostate cancer in Sweden using a prevalence-based cost-of-illness approach. Resource utilisation and related costs within Stockholm Region during 2016 were quantified using data from the Stockholm PSA and Biopsy Register and other health and population registers. Costs included: (i) direct medical costs for health care utilisation at primary care, hospitals, palliative care and prescribed drugs; (ii) informal care; and (iii) indirect costs due to morbidity and premature mortality. The resource utilisation was valued using unit costs for direct medical costs and the human capital method for informal care and indirect costs. Costs for the Stockholm region were extrapolated to Sweden based on cancer prevalence and the average costs by age and resource type.

**Results:**

The societal costs due to prostate cancer in Stockholm in 2016 were estimated to be €64 million Euro (€Mn), of which the direct medical costs, informal care and productivity losses represented 62, 28 and 10% of the total costs, respectively. The total annual costs extrapolated to Sweden were calculated to be €281 Mn. The average direct medical cost, average costs for informal care and productivity losses per prevalent case were €1510, €828 and €271, respectively. These estimates were sensitive to assumptions related to the proportion of primary care visits associated with PSA testing and the valuation method for informal care.

**Conclusion:**

The societal costs due to prostate cancer were substantial and constitute a considerable burden to Swedish society. Data from this study are relevant for future cost-effectiveness evaluations of prostate cancer screening and treatment.

## Background

Globally in 2018, prostate cancer (PCa) was the second most frequent cancer diagnosed and the fifth leading cause of cancer death in males [1]. In Sweden, the availability of prostate-specific antigen (PSA) testing has led to increased incidence rates and a gradual decline in mortality rates [2, 3]. However, PCa is the most common cause of cancer death among Swedish men [4, 5]. One consequence of the combination of increased incidence, long lead-times associated with testing, and good survival for localised PCa is that the prevalence of PCa has increased markedly. In 2016, approximately 25,000 and 110,000 males were living with a diagnosis of PCa in Stockholm and Sweden [5], accounting for 2.3 and 2.1% of the male population, respectively.

Although PSA has been commonly used as a screening test for PCa [6], the balance between the benefits and harms of PSA testing has been debated. The European Randomized Study of Screening for PCa found a  mortality reduction of 20% after 16-year follow-up from PSA testing compared with no testing [7]. However, PSA testing is also associated with potential harms, including unnecessary biopsies, over-diagnosis of low-risk cancers and over-treatment [4, 5]. Unnecessary biopsies and over-diagnosis may not only reduce health-related quality of life of the patients, but they are also associated with increased costs due to increased health-care visits, biopsy-related complications, over-treatment and lost productivity [4].

Complementary diagnostic tools may reduce the harms and increase the health benefits from early detection. In recent years, risk assessment using Magnetic Resonance Imaging (MRI) together with MRI-targeted biopsies has improved specificity and sensitivity for high risk PCa [8, 9]. Although the cost of prostate MRI has declined with increased use of abbreviated MRI protocols, using MRI with targeted biopsies is associated with increased costs compared with traditional biopsy procedures.

For the treatment of low-risk PCa, active surveillance (AS) has been recommended by Swedish clinical guidelines since 2007 [10]. For intermediate- and high-risk PCa patients, radical prostatectomy (RP) and radiation therapy (RT) are common treatment modalities. In 2016, 98.1% of all RP procedures in public hospitals were robot-assisted in Stockholm [11], which are associated with 10–42% higher costs than open surgery [12]. For localised high-risk patients, a combination of RT and adjuvant androgen deprivation is mostly used with curative intent. For recurrent and metastatic PCa patients, the first-line treatment is hormone therapy (or androgen deprivation therapy). Two recent drugs, Abiraterone and Enzalutamide, have a newer mechanism of action and a more convenient form of administration; however they are associated with high costs.

Previous cost-of-illness (COI) studies performed in Sweden estimated that the total costs due to PCa for the years 1985, 1993 and 1998 were 51 million Euro (€Mn), €86Mn and €109Mn (not adjusted by the consumer price index (CPI)) respectively, with a 10% annual increase between 1985 and 1998. However, these studies were based on small cohorts and only considered health care utilisation [13–16].

Costs due to PCa from a societal perspective have been estimated in two studies in the past decade. In a European population-based cost study from 2009 for major cancer sites, the total cost for PCa in Sweden was estimated to be €237Mn [17]. However, many of the calculations for Sweden were based on assumptions or data from other countries or using European level data [17]. In a 2013 report in Swedish, the costs were estimated to be €321Mn [18]. This study employed data from multiple health registers, with assumptions from clinical experts as well as a regional price list. However, in both studies, details and assumptions were incompletely reported, including the diagnosis-related groups (DRGs) that were used to calculate inpatient and outpatient costs, a full drug list for treating PCa, separate costs of the prescribed (pharmacy-based) and requisition (hospital-based) drugs, as well as unit costs for each type of resource.

This study aimed to (i) estimate the annual economic burden of PCa in the Stockholm region and Sweden in 2016 and (ii) provide reference values for future cost-effectiveness analyses of PCa screening and treatment.

## Methods

As per economic evaluations for the Swedish Dental and Pharmaceutical Benefits Agency (TLV) [19] and recommendations from previous studie, all costs to society should be taken into account when conducting a cost-of-illness study for making decisions about the resource allocation [20–22]. In this prevalence-based cost-of-illness study, the total annual cost of PCa was estimated using a societal perspective. We used a bottom-up approach to estimate the economic burden [23]. Direct and indirect costs during 2016 were estimated in three steps, including (i) the identification of categories of resource use, (ii) quantification of resources use, and (iii) valuation of the identified resources [24]. The total costs for PCa in Stockholm were calculated by multiplying the identified quantities of resource use with the unit costs of each resource. Costs were adjusted for inflation by using the CPI [25] and converted to 2016 Euros (mean annual exchange rate €1 = 9.47 Swedish Krona) [26]. To calculate a national cost, the results were extrapolated to males in Sweden. Data were analysed using SAS version 9.4.

### Study population

The study population included all males residing in the Stockholm region at the end of 2015 using the Stockholm PSA and Biopsy Register (SPBR), which has linked all PSA tests and prostate biopsies from the laboratories serving the Stockholm region to multiple health and population registers in Sweden. For the biopsies, this includes both negative biopsies and biopsies indicating PCa. Those who have left Stockholm Region during 2016 would have under-reported PSA tests and biopsies. The linked registers include: the National Prostate Cancer Register; the National Cancer Register; the Total Population Register; the National Patient Register, including inpatient and outpatient events; the National Death Register; and the National Prescribed Drug Register (PDR) [27].

### Direct medical costs

#### Inpatient and outpatient care

By using the SPBR, the numbers of events from *inpatient care and outpatient care* were identified using the Nordic Diagnostic Related Groups Swedish DRG classification system. The International Classification of Diseases, 10th version (ICD-10) code C61.9 was used to identify cases related to PCa as the primary diagnosis together with Swedish DRG codes associated with PCa related events. For details of the event identification, see Appendix A. The unit costs for each DRG were extracted from the Stockholm region price list [28]. The costs for inpatient and outpatient care were calculated by the number of DRGs multiplied by the unit costs.

#### Primary care

Resources utilised in primary care were based on PSA tests recorded from the SPBR. For each PSA test, if the sample date was not performed during inpatient care and not on the same date as an outpatient care, it was considered as being conducted in primary care. Furthermore, PSA tests undertaken prior to a diagnosis of PCa were categorised as diagnostic tests, and PSA tests undertaken on the same date or after the diagnosis of a PCa were categorised as monitoring tests. A large proportion of primary care visits for patients who had a PSA test may not be associated with PCa testing alone. As per an earlier report, we assumed that 20% of the consultation cost with a PSA test was associated with PCa testing [29]. The average unit cost of a primary care visit was based on data for the Stockholm region in 2014 from the National Board of Health and Welfare (NBHW) [30] and the growth rate of the unit cost from the Southern Health Region’s price lists in 2015 and 2016 [31, 32]. For PSA tests, a test analysis cost was added to the costs for the primary care visit [33]. The costs for primary care were calculated by the number of visits due to diagnostic and monitoring testing, multiplied by the unit costs.

#### Pharmaceutical costs

Thirteen *prescribed drugs* and hospital-based *requisition drugs* (substances, see Appendix B1 for a drug list) were used in Sweden for treating PCa [34–37]. We extracted aggregated costs at the fifth level of the Anatomical Therapeutic Chemical Classification System (ATC5) from the Concise Database of the Swedish eHealth Agency [37] for the Stockholm region. For *prescribed drugs* with multiple indications, we used the PDR to identify the proportion of drug uses by males aged 18 and above [37] and the SPBR to estimate the proportion of drug uses for PCa. For *requisition drugs* used for multiple indications, data from the Stockholm Electronic Patient Records (SEPR) Corpus Health Bank from Stockholm University [38, 39] were extracted to estimate PCa associated costs. Counts of drug use by brand, age and sex were multiplied by the unit costs from The Dental and Pharmaceutical Benefits Agency (TLV) drug database [40]. Appendix B2 illustrates the steps in the cost calculations.

#### Palliative care

To estimate the costs of palliative care due to PCa, data from the Swedish Register of Palliative Care (SRPC) were linked to the SPBR. The data in SRPC were primarily collected from an end-of-life questionnaire, which contains 30 questions usually completed by healthcare staff after a patient’s death [41]. In the questionnaire, the “place of death” indicated the type of care that a patient received [41]. As palliative care inside the hospital ward was captured by inpatient care (see Appendix C1), we restricted data to the care taken place at the hospice/palliative inpatient care, the nursing home, or the patient’s home. The total number of days in each type of palliative care and the total number of patients were calculated. The unit costs per day in each type of care were extracted from existing studies [42–44] and calculated to year 2016 using CPI. The total costs due to palliative care were calculated by summing up the products of the total number of care days and the cost per day of each type of care. See Table 2 and Appendix C2 for details.

### Informal care

The measurement of resource use (numbers of hours) for informal care was conducted using existing and published data from WAVE2 and WAVE3 of the Survey of Health, Ageing and Retirement in Europe (SHARE) project [47]. The participants in WAVE2 were asked whether they were severely limited in daily activities and other questions regarding the care received inside or outside the household. In WAVE3, the proxy respondents such as partner, child or other relationship with the deceased person answered a series of questions regarding the care provided to the patients in the last 12 months before their deaths. For details of the proxies, see Appendix D2. We used the information from WAVE2 and WAVE3 to estimate the age-specific hours of informal care for *patients severely limited in daily activities* and *patients that were terminally ill.* Logistic regressions were applied to estimate the probability of being severely limited in daily activities and the probability of receiving informal care due to cancer. Linear regressions were used to estimate the hours of care received and the probability of caregivers at working age. See Appendix D1–6 for details. We assumed caregivers at working age provided care during work hours and followed similar assumptions to those used in the European study and a Swedish report [17, 18] to decide what type of caregiver is at working age (see Appendix D4 and D6). Following recommendations for health economic evaluations, we applied the annual general gross wage rate for all working individuals of both genders [24, 50], which was €56,930 including social security contributions (36.98%) [25, 51] in 2016. Based on 253 workdays and an 8-h work day (full time), the cost per hour was estimated to be €28.1. The total costs for informal care were multiplied by the estimated hours of care and cost per hour.

### Productivity losses

We used the human capital method to estimate the costs following a diagnosis of PCa related to *lost productivity due to morbidity (sick leave and early retirement)* and *mortality (premature death)*. We valued lost productivity in terms of gross earnings [52] and assumed full employment through to the general retirement age 65 years in Sweden. Using the average annual gross earnings for both genders in Sweden in year 2016, the cost of a full work day was estimated to be €225.0.

#### Productivity losses due to morbidity

The number of men with a primary cancer diagnosis code of ICD-10 C61 during 2016 in Stockholm and the number of days on long-term sick leave (more than 14 days) and early retirement were retrieved from the Swedish Social Insurance Agency (Försäkringskassan, abbreviated FSK). The diagnosis code used by FSK was primarily based on a medical certificate completed by a general practitioner. The men identified from FSK were linked to the cohort using anonymised IDs. In Sweden, individuals can receive 100, 75, 50% or 25% cash benefit if a *long-term sick leave* is taken. *Short-term sick leave* (14 days or less) is covered by the employer and is not reported to FSK. For patients who had long-term sick leave, we assumed that a 100% short-term sick leave was taken. For people aged 30–64 years, a sickness compensation can be granted if their work capacity is permanently impaired and proportional compensation also applies to *early retirement* [53]. To estimate the cost of lost productivity due to morbidity, the total number of net workdays lost due to sick leave and early retirement was multiplied by the average daily income for full-time employees.

#### Productivity losses due to premature death

We extracted the number of males that died from PCa during 2016 in Stockholm and their age of death from the SPBR. For each deceased male, the accumulated losses of years were calculated by integrating the population-based survival rate from the patient’s age of death through to age 65 years [54]. Costs for future productivity losses were discounted at 3% yearly in accordance with international and Swedish recommendations [19, 50]. See Appendix E for details on these calculations.

### Extrapolation to Sweden

The annual costs due to PCa in Stockholm were extrapolated to Sweden based on the average annual costs per prevalent case for each type of resource in Stockholm by 10-year age groups multiplied by the number of prevalent cases in Sweden in each age group extracted from Nordcan [5]. For productivity losses due to premature mortality, the average cost per death was multiplied by the number of PCa deaths in Sweden. See Appendix F for the pattern of prevalence, incidence and mortality in Stockholm and Sweden through to 2016.

### Sensitivity analyses

Sensitivity analyses were performed to address the uncertainty in key parameters. *First*, records show that 87% of cancer deaths in Sweden were reported to SRPC in 2015 [41]. Therefore, the potential *palliative care* for the PCa patients who were recorded in the SPBR but not reported to the SRRC in 2016 was considered in a sensitivity analysis (see Appendix C2). *Second*, *primary care* of men who had PSA tests without diagnoses of PCa were not considered in the European study and the Swedish report. The corresponding costs were excluded in a sensitivity analysis. *Third*, we estimated the costs for *informal care* using the proxy good method, which values the care at a market price, considering the care would have been provided by a formal caregiver [50, 55–58]. We applied the hourly cost at a nursing home as a proxy at €26.1 [42] to the total hours calculated, irrespective of whether the care was provided by someone at a working age or not. *Lastly*, the *unit cost of prostate biopsies* from the price list in the base case may be lower than the cost to a clinical department. A unit cost at €1159 (Biopsy at outpatient care: w/o MRI, Table 4) was used to investigate the effect of this uncertainty.

### Illustration and costs for different diagnosis and treatment pathway

Resource use in the diagnosis phase, active surveillance, treatment phase and post-treatment follow-up was described for the clinical guidelines in Sweden [59] and estimated for standardised pathways.

## Results

We present (i) detailed results for the Stockholm region, (ii) aggregated results for Sweden, (iii) sensitivity analyses and (iv) a description of costs by treatment pathways.

### Prevalence, incidence and mortality in the Stockholm region

In total, there were 1772 incident cases of PCa in 2016, which were 7.0% of the 25,490 prevalent patients (Table 1). Of the 995 prevalent cases who died in 2016, 38.8% died from PCa. In summary, PCa was uncommon before the age of 50 years, incidence rates were highest among those aged 70–79 years, and prevalence was highest among those aged 80–89 years. Mortality rates for PCa increased rapidly with increasing age.

**Table 1:** Description of the study population by 10-year age group, Stockholm Region, 2016

Age (Years)	Men in Stockholm		Age-specific Prevalence-PCa		Incidence-PCa		Mortality-all cause		Mortality-PCa	
	No.	% of Total	No.	% by age	No.	Rate per 1000	No.	Rate per 1000	No.	Rate per 1000
0–9	130,411	12.0%		0.0%		0.00		0.00		0.00
10–19	116,537	10.7%		0.0%		0.00		0.00		0.00
20–29	140,326	12.9%		0.0%		0.00		0.00		0.00
30–39	169,731	15.6%		0.0%		0.00		0.00		0.00
40–49	163,090	15.0%	134	0.1%	31	0.19		0.00		0.00
50–59	141,519	13.0%	1868	1.3%	252	1.78	6	0.04	6	0.04
60–69	108,005	10.0%	7297	6.8%	601	5.56	87	0.81	43	0.40
70–79	79,506	7.3%	11,079	13.9%	692	8.70	289	3.63	122	1.53
80–89	29,330	2.7%	4365	14.9%	176	6.00	420	14.32	148	5.05
90+	6605	0.6%	747	11.3%	20	3.03	193	29.22	67	10.14
Total	1,085,060	100.0%	25,490	2.3%	1772	1.63	995	0.92	386	0.36

### Direct medical costs

#### Inpatient and outpatient care

Ten PCa-related DRGs were identified for 1602 patients from inpatient care (Table 2); 45.8% of those patients were aged 70–79 years. Radical prostatectomy was the most frequent DRG with the highest cost of over €6.9Mn, contributing to 66.4% of the total costs for *inpatient care*. We identified 4841 patients with resource utilisation in outpatient care from eight DRGs (Table 2). Over 60% of the costs of *outpatient care* were associated with radiation therapy, with an annual estimated cost of more than €6.3Mn. Of the 3956 episodes of prostate biopsies (N75O, Table 2), approximately 45% were undertaken as diagnostic biopsies (that is, with no prior prostate cancer diagnosis) and 55% were conducted after a prostate cancer diagnosis.

**Table 2:** Unit costs and quantity of resource utilization due to PCa, Stockholm region, 2016 (€)

**Inpatient care - by DRG**	**No. Patients**	**No. Episodes**	**Source**	**Unit cost/Case (€)**	**Source**
N01N Radical prostatectomy	647	647	SPBR	10,730	Price list from Stockholm region [28]
N05N Transurethral resection of prostate	57	57		4269	
N10C Testes malignancy, operation (OR) procedures, with complications	8	8		6051	
N30C Reproductive system malignancy, OR procedures, with complications	5	5		6001	
N30E Reproductive system malignancy, OR procedures	4	6		6001	
N40C Reproductive system malignancy, other procedures, with complications	198	253		5647	
N40E Reproductive system malignancy, other procedures	52	55		4186	
N05N Transurethral resection of prostate (Non-primary)	16	16		4269	
R40C Radiation therapy, with complications (Non-primary)	64	92		7590	
R40E Radiation therapy (Non-primary)	76	118		6206	
**Outpatient care - by DRG**	**No. Patients**	**No. Episodes**	**Source**	**Unit cost/Case (€)**	**Source**
N32O Reproductive system malignancy, OR procedures	3	3	SPBR	814	Price list from Stockholm region [28]
N40O Reproductive system malignancy	813	1929		482	
N75O Biopsy, male genitalia, including consultation	426	436		664	
N99X Team consultation for diseases of male genitalia	135	214		476	
N99O Specialist consultation for diseases of male genitalia (Non-primary)	31	39		388	
X11O Radiation therapy, resource-intensive (Non-primary)	381	9650		515	
X12O Radiation therapy, including preparatory measures (Non-primary)	568	624		415	
X14O Radiation therapy, less resource-intensive (Non-primary)	286	4499		249	
**Primary care**	**No. Patients**	**No. Visits**	**Source**	**Unit cost/Visit (€)**	**Source**
GP visit - Diagnostic testing	82,066	101,041	SPBR	74	NBHW, Price list of Stockholm and Southern region [30–32]
GP visit – Monitoring testing	18,908	39,481		74	
**Palliative care**	**No. Patients**	**No. Days**	**Source**	**Unit cost/Day (€)**	**Source**
Hospice/palliative inpatient care	127	2257	SRPC [45]	722	[42–44]
Home support - daily contact of home service	1	225		13	[44]
Home support - Specialised home-care team	52	3006		355	[42, 43]
Nursing home – permanent	37	4618		209	[42]
Nursing home - short-term	11	400		209	[42]
**Pharmaceuticals*(Prescribed)*- by substance**	**No. Patients**		**Source**	**Mean cost/Patient (€)**	**Source**
L01CD02 – Docetaxel	2		SPBR	1174	SPBR, PDR [46] TLV [40]
L01CD04 – Cabazitaxel	0				
L01DB07 – Mitoxantrone	0				
L02AA02 – Polyestradiol phosphate	89			831	
L02AE01 – Buserelin	22			565	
L02AE02 – Leuprorelin	2102			1138	
L02AE03 – Goserelin	318			1792	
L02AE04 – Triptorelin	454			640	
L02BB01 – Flutamide	81			231	
L02BB03 – Bicalutamide	3098			184	
L02BB04 – Enzalutamide	264			16,984	
L02BX02 – Degarelix	4			326	
L02BX03 – Abiraterone	78			15,416	
**Informal care**	**No. Patients**	**No. Hours**	**Source**	**Mean salary/Hour (€)**	**Source**
Severely limited in daily activity - outside	2530	495,558	SHARE [47], SPBR	28	SCB [48]
Severely limited in daily activity - inside	439	98,979		28	
Terminally ill	153	49,697		28	
**Productivity losses - morbidity**	**No. Patients**	**No. Days**	**Source**	**Mean salary/Day (€)**	**Source**
Short-term sick leave	265	17,893	FSK	225	SCB [48]
Long-term sick leave	7	1649		225	
**Productivity losses - pre-mature mortality**	**No. Patients**	**No. Years**	**Source**	**Gross earning/Year (€)**	**Source**
Pre-mature mortality	17	4.32	SPBR, NBHW [49]	56,930	SCB [48]

*DRG* Diagnosis related group, *FSK* Swedish Insurance Agency, *GP* General Practitioner, *NBHW* National Board of Health and Welfare, *OR* operation, *PDR* Prescribed Drugs Register, *SCB* Statistics Sweden, *SHARE* Survey of Health, Ageing and Retirement in Europe, *SPBR* Stockholm PSA and Biopsy Register, *SRPC* Swedish Register of Palliative Care, *TLV* Dental and Pharmaceutical Benefits Agency

#### Primary care

Costs directly associated with PSA testing accounted for 7.5% of the total costs for PCa. Approximately 73% of the PSA tests were undertaken as diagnostic testing. On average, PCa patients conducted 2.0 PSA tests for monitoring the disease in 2016 (Table 2).

#### Pharmaceutical costs

Of the 13 substances listed (Table 2), Bicalutamide was the most frequently prescribed drug, used by approximately 3100 patients (Table 2). Cabazitaxel accounted for over half of the total requisition drug costs (€1.1Mn). The antiandrogens Enzalutamide and Abiraterone had an annual cost of €4.8Mn and €1.2Mn, respectively, accounting for 57% of the total drug costs due to PCa in the Stockholm region in 2016.

#### Palliative care

Among the 386 deaths due to PCa in 2016 (Table 1), 267 deaths were reported to SRPC. Of these patients, 228 received palliative care other than hospital inpatient care (Table 2) with an average direct cost of €16,441 per patient. The patients who died in hospice or palliative inpatient care stayed on average for 18 days. Patients with home support by a specialised home-care team had an average length of 58 days. Nursing home based patients had an average of 125 and 36 days with permanent or short-term stays, respectively.

### Informal care

For patients who were *severely limited in daily activities*, informal care was primarily provided by relatives or friends outside the household (83%), with approximately 1 h per day of the help. This value reduced to 0.5 h per day when restricted to caregivers aged less than 65 years. The average time for informal care provided by someone inside the household was approximately 1.1 h per day, of which 0.6 h per day were provided by caregivers aged less than 65 years. For patients who were *terminally ill*, almost 4 h of informal care were provided per day, of which approximately 1 h care per day was provided by caregivers aged less than 65 years.

### Productivity losses

Productivity losses due to *sick leave* and *early retirement* were estimated to be €2.8Mn and €0.3Mn (Table 3). The 265 patients aged 40–64 years were on sick leave for an average of 68 days per person (Table 2), of which 14 days were short-term sick leave paid by the employers. Patients who retired early due to PCa took disability pension for an average of 236 days per person. Seventeen men aged less than 65 years died of PCa in the Stockholm region during 2016(Table 2). Productivity losses due to *premature mortality* were estimated to be €3.6Mn (Table 3).

**Table 3:** Costs due to PCa by type of resource, Stockholm Region and Sweden, 2016 (€)

Type of resource	Stockholm Region		Sweden	
	Costs (€)	Costs (%)	Costs (€)	Costs (%)
**Healthcare related costs**	**39,765,502**	**61.6%**	**162,462,861**	**57.9%**
Inpatient care	10,457,640	16.2%	41,041,967	14.6%
Outpatient care	10,025,188	15.5%	40,450,037	14.4%
Primary care	4,810,643	7.5%	17,782,058	6.3%
Palliative care	3,748,553	5.8%	17,914,185	6.4%
Pharmaceuticals	10,723,478	16.6%	45,274,615	16.1%
**Informal care**	**18,120,816**	**28.1%**	**89,142,341**	**31.7%**
**Productivity losses**	**6,626,929**	**10.3%**	**29,176,618**	**10.4%**
Morbidity – Sick leave	2,783,210	4.3%	8,534,334	3.0%
Morbidity – Early retirement	256,420	0.4%	806,630	0.3%
Pre-mature mortality	3,587,299	5.6%	19,835,654	7.1%
**Total**	**64,513,247**	**100%**	**280,781,820**	**100.0%**

### Total costs and extrapolation to Sweden

The total annual costs due to PCa were estimated to be €65Mn in Stockholm region and €281Mn in Sweden (Table 3). The cost per capita in Stockholm was estimated to be €59, which was higher than the estimated cost at €56 per male in Sweden. This was partially explained by a higher PCa prevalence per capita in Stockholm. The largest share of costs were related to health care (57.9%) followed by productivity losses (31.7%) and informal care (10.4%).

### Sensitivity analyses in the Stockholm region

**Fig. 1:**
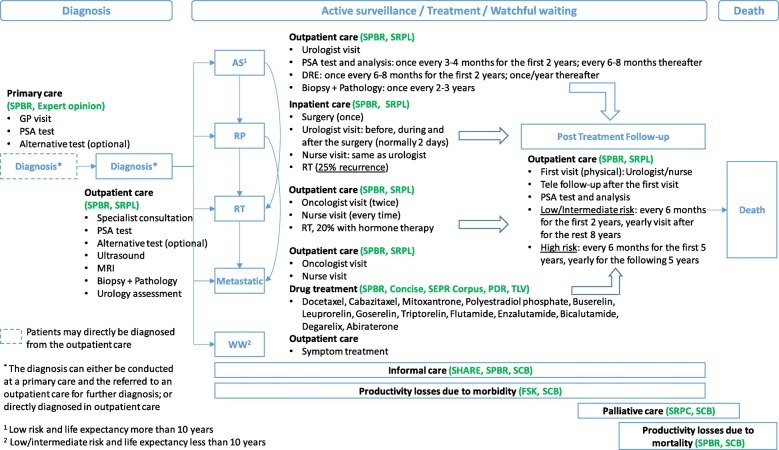
Simplified diagnosis and treatment pathways of patients diagnosed with prostate cancer. AS: Active Surveillance; DRE: Digital Rectal Examination; FSK: The Swedish Social Insurance Agency; GP: General Practitioner; MRI: Magnetic Resonance Imaging; PDR: Prescribed Drug Register; RP: Radical Prostatectomy; RT: Radiation Therapy: SCB: Statistics Sweden; SEPR Corpus: Stockholm Electronic Patient Records Corpus; SHARE: Survey of Health, Ageing and Retirement in Europe; SPBR: Stockholm PSA and Biopsy Register; SRPC: Swedish Register of Palliative Care; SRPL: Stockholm Region Price List; TLV: The Dental and Pharmaceutical Benefits Agency; WW: Watchful Waiting

Sensitivity analyses led to a − 5.5 to + 24.7% change of the total costs in Stockholm in 2016 (Fig. 1). Excluding costs in the primary care setting for men with no prior PCa diagnosis, the total costs decreased markedly by 5.5%. Using the proxy good method to value the informal care doubled the costs for informal care and increased the total costs by 24.7%. Including the costs for those not reported to SRPC and altering the biopsy cost showed minor differences in the total costs.

### Illustration and costs for different treatment pathways

**Fig. 2:**
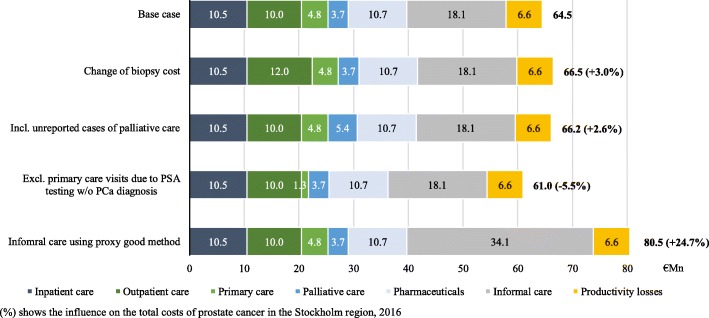
Sensitivity analyses of costs due to prostate cancer in the Stockholm region (€Mn). (%) shows the influence on the total costs of prostate cancer in the Stockholm region, 2016

Resource use and the frequency of the resource use for diagnosis, AS, RP, RT, treatment for patients with metastatic PCa, and watchful waiting (WW) are summarised in Fig. 2. The average costs for different treatment pathways are summarised in Table 4. If a patient was referred to outpatient care for a biopsy, the total costs when employing an additional MRI-guided biopsy increased the costs of a biopsy by over 30% (€1513), compared with using a systematic biopsy (€1159). Similarly, use of MRI under AS increased the annual costs by at least 20% (€704). The annual treatment costs for patients with metastatic PCa by using both chemotherapy and hormone therapy (€7283) showed minor differences to using hormone therapy only (€6867).

**Table 4:** Costs of managing prostate cancer by treatment pathway, 2016 (€)

Module/Procedure	Unit cost (€) base/alternative	Resource use base/alternative	Cost/patient (€) base/alternative	Source/Remarks
**Diagnosis**				
PSA test at primary care				
GP visit	152	0.2	30	[29–32, 72]
PSA test analysis	4	1	4	[33]
**Total costs/patient**			34	
Biopsy at outpatient care: w/o MRI				
Specialist and nurse consultation	147	1	147	[73]
Ultrasound	151 / 212	1	151 / 212	[74] / [73]
Biopsy	304	1	304	[33]
Pathology	516	1	516	[73]
Nurse consultation	40	1	40	[73]
**Total costs/patient**			1159 / 1220	
Biopsy at outpatient care: with MRI				
Specialist and nurse consultation	147	1	147	[73]
Ultrasound	151 / 212	1	151 / 212	[74] / [73]
MRI	354 / 569	1	354 / 569	[73] / [74]
Biopsy	304	1	304	[73]
Pathology	516	1	516	[33]
Nurse consultation	40	1	40	[73]
**Total costs/patient**			1513 / 1789	
**Treatment**				
Active surveillance at outpatient care: w/o MRI				
Specialist and nurse consultation	147	1	147	[73]
PSA test sampling	36	1	36	[29]
PSA test analysis	4	3 / 4	11 / 15	[33]
Ultrasound	151	0.33 / 0.5	50 / 76	[74]
Biopsy	304	0.33 / 0.5	100 / 152	[73]
Pathology	516	0.33 / 0.5	170 / 258	[33]
**Total costs/patient**			587/ 792	***Annual cost***
Active surveillance at outpatient care: with MRI				
Specialist and nurse consultation	147	1	147	[73]
PSA test sampling	36	3 / 4	108 / 144	[29]
PSA test analysis	4	3 / 4	11 / 15	[33]
Ultrasound	151	0.33 / 0.5	50 / 76	[74]
MRI	354 / 569	0.33 / 0.5	117 / 285	[73] / [74]
Biopsy	304	0.33 / 0.5	100 / 152	[73]
Pathology	516	0.33 / 0.5	170 / 258	[33]
**Total costs/patient**			704 / 1077	***Annual cost***
Radical prostatectomy at inpatient care: open surgery				
Open surgery	7422	1	7422	[12]
Specialist and nurse consultation	147	1 / 2	147 / 295	[73]
RT	672	0.25	168	[12]
**Total costs/patient**			7738 / 7885	
Radical prostatectomy at inpatient care: robot-assistant				
Robot assisted surgery	9941 / 12,328	1	9941 / 12,328	[12]
Specialist and nurse consultation	147	1 / 2	147 / 295	[73]
RT	672	0.25	168	[12]
**Total costs/patient**			10,257 / 12,791	
Radiation therapy at outpatient care				
Oncologist - new visit	397	1	397	[32]
Oncologist - further visit	171	1	171	[32]
Nurse consultation	40	20	808	[73]
RT	672	20	13,440	[12] [75]
Hormone therapy-annual	6867	0.20	1373	
**Total costs/patient**			16,189	
Metastatic: Chemo + Hormone therapy				
Annual cost/patient	7283	1	7283	Results of study
**Total costs/patient**			7283	***Annual costs***
Metastatic: Hormone therapy				
Annual cost/patient	6867	1	6867	Results of study
**Total costs/patient**			2136	***Annual costs***
**Post treatment follow-up**				
Post treatment follow-up: Low/intermediate risk				
Specialist and nurse consultation	147	1	147	[73]
Specialist consultation - Tele follow-up	15	11	162	[73]
PSA test sampling	36	12	432	[29]
PSA test analysis	4	12	46	[33]
**Total costs/patient**			788	
Post treatment follow-up: High risk				
Specialist and nurse consultation	147	1	147	[73]
Specialist consultation - Tele follow-up	15	14	206	[73]
PSA test sampling	36	15	541	[29]
PSA test analysis	4	15	57	[33]
**Total costs/patient**			952	

*GP* General Practitioner, *MRI* Magnetic Resonance Imaging, *PSA* Prostate-specific Antigen, *RT* Radiation Therapy

## Discussion

### Main findings

This study is the first to investigate the economic burden of PCa in Stockholm from a societal perspective. The annual cost of PCa in Sweden during 2016 was estimated to be €281Mn. *Direct medical costs, informal care* and *productivity losses* accounted for approximately 58, 32 and 10% of the total burden, respectively.

### Comparisons with existing evidence and methodological considerations

Our estimates of the total economic burden are higher than the results from a previous European study [17]. This discrepancy can, in part, be explained by the different estimation methods of the *primary care* costs; our estimate is more than eight times higher than the costs for primary care in the European study. We estimated the primary care resource utilisation based on the number of visits due to PSA tests, of which 73% were found to be not associated with a previous PCa diagnosis. In the European study, resource utilisation of primary care was calculated based on the overall number of visits, the proportion of visits due to all cancers and the proportion of visits due to PCa [17]. The proportion of visits due to call cancers was estimated from a sample of 26 GPs using electronic health records from Stockholm [60], while the proportion of visits due to PCa was evaluated indirectly using the proportion of hospital discharge due to PCa out of all cancers [17], which may be biased.

In the European study, costs for accident and emergency care accounted for up to 2% of the total costs for PCa among all 27 countries [17]. Due to the lack of data, Danish data were used to estimate those costs in Sweden [17]. PCa is less related to the accident and emergency care, while palliative care was measured in our study, where the latter accounted for 6% of the total costs. Including PCa patients not recorded by SRPC in a sensitivity analysis, the costs for palliative care reached 8% of the total costs in Stockholm.

Methodologically, there is a potential gap between the hospital care costs calculated by DRGs and the actual costs of treatment episodes from the hospital departments, particularly for prostate biopsies in the *outpatient* setting. The unit cost of a prostate biopsy including the physician visit based on DRG N75O (€664) is lower than that from the hospital departments (€1159 – €1789). This may be explained by several reasons. First, the DRG cost is based on the average cost per DRG code, which is calculated from the average cost for all care occasions. To control the budget, often there is a ceiling for the quantity of DRG points to be produced by each caregiver. If the ceiling is exceeded, less compensation will be given per point produced [61]. In summary, if the quantity of N75O used is more than planned, the compensation for each N75O could be lower than its real costs. Second, many regions in Sweden have lessened the importance on DRG as a reimbursement model, which possibly resulted in less accuracy in recording DRGs in the diagnoses and treatments [61, 62].

Ten percent of *drug costs* for treating PCa were hospital-based requisition drugs, which is lower than the average proportion of requisition drugs of the total cost of cancer drugs at 54% [37]. This could partly be explained by the uptake of Enzalutamide since its introduction to Sweden in 2014. Enzalutamide accounted for 45% of the total PCa drug costs in Stockholm during 2016 and had a 97% of its sales as prescribed drugs. Other drugs, dominated by the costs in the hospital, had very low sales in 2016. In addition, many cancer drugs are used for multiple indications, including non-cancer diseases. For the 13 substances treating PCa, almost half were used for other indications. Due to limited data availability, we used data in 2012 from the PDR and data in 2014 from SEPR Corpus to obtain the proportion of usage by the indication of PCa. Given the increased prevalence of PCa, there is a need to better characterise these costs by source of utilisation and by indication.

Comparing the proportion of costs for *productivity losses* with cost analyses of other disease areas such as depression (65%), breast cancer (70%), multiple sclerosis (79%) and brain tumors (74%) [63–66], the costs for productivity losses due to PCa accounted for a much lower share of the total costs. This pattern may be mainly explained by most PCa patients being over age 65 years. The estimated costs of *productivity losses due to morbidity* in 2016 were lower compared with estimates from the European study for 2009 [17]. The lower estimates can possibly be explained by a reduction in the average days of sick leave compensation from 90 days [67] in 2009 to 72 days in 2016 [53] of men in Sweden, or by a considerable decrease in the number of men compensated for early retirement by 36% from 2009 to 2016 [53, 67]. Furthermore, the estimated costs of *productivity losses due to premature mortality* in 2016 were also lower than the costs in 2009. This may be explained by the fact that the European study assumed 79 years old as the age of retirement for all countries [17], which is 14 years later than the general age of retirement in Sweden. In addition, it can also be partly explained by an absolute reduction in the number of PCa deaths below age 65 years from 138 in 2009 to 94 in 2016 [49].

We used the human capital approach to estimate the value of the productivity losses. A common criticism of this approach is that it discriminates against those elderly individuals who are not within a working age. One may argue that patients at age 65 years or over would produce housework, babysitting of grandchildren, voluntary work or other unpaid productivity [22]. This criticism is especially relevant when estimating indirect costs for PCa, since the majority of PCa patients are of retirement age. An alternative way of measuring the productivity losses is the friction cost method [24]. This approach, which measures the productivity loss due to an absent worker, provides a lower bound on these values compared with the human capital method. The friction cost method has been criticised since it does not consider the replacement cost for an absent worker or the loss in team productivity [68, 69]. Note that this method also does not include patients aged 65 years and over.

Informal care was measured in terms of productivity losses of the caregiver prior to age 65 years as an opportunity cost, which is the most commonly used method in measuring costs for informal care [55, 70]. It could be argued that all caregivers, irrespective of their age, possibly used leisure time to provide care. There was a lack of data on whether the caregivers reduced their work time to provide informal care. However, leisure time is usually difficult to value and can be valued as being zero, as rates reflecting “home pay” or the market value for caregivers, or as overtime earnings [50, 57, 58]. We used the proxy good method to value informal care in a sensitivity analysis. The unit cost of nursing services was applied as a proxy and was similar to the general population. However, the estimation of care-time was considerably higher. Other researchers have observed that these two methods can yield widely varying estimates of care-time [70, 71].

It should be noted some costs due to palliative care, informal care and productivity losses can be associated with comorbidities, such as chronic diseases or concurrent diagnoses of other cancers. In this study, palliative care and productivity losses due to mortality were assigned based on primary cause of death. Informal care and productivity losses due to morbidity were assigned based on primary cancer diagnosis. It can be argued that PCa patients with comorbidities could lead to reduced PCa-related costs, because PCa patients might receive palliative care such as relieving pains caused by multiple diseases or take sick leave due to multiple diagnoses.

### Strengths and limitations

To our knowledge, this is the first COI study of PCa in Stockholm. This study has a number of strengths. *First*, it provides a detailed description of resource utilisation and unit costs using the linked Swedish health and population registers. In particular, we carefully characterised the treatment pathways by resource type and the frequency of resource utilisation for combinations of diagnostic tests and treatment modalities for PCa patients. *Second*, this study provides all substances and reported the costs of both prescribed and requisition drugs treating PCa patients. This improves on earlier COI studies for Sweden [17, 18]. *Third,* the PSA data from SPBR linked with PCa diagnosis allowed for a population-based description of PCa testing in the primary care setting. *Fourth*, the productivity losses were calculated using a general annual gross earnings for both genders during 2016 in Sweden to account for equity issues. *Finally*, the sensitivity analyses reflect how the uncertainties may have affected the results.

Some limitations should also be noted. *Firstly*, extrapolating data on PCa prevalence and costs from the Stockholm region to the national level could be less reliable. However, the general patterns for PCa incidence and mortality in Stockholm region are similar to the national averages, the guidelines for prevention and treatment of PCa are national, and we have indirectly adjusted for differences in PCa testing by the adjustment for PCa prevalence. *Second*, there is no nationally representative price list and the reported DRG unit costs for Stockholm and Sweden may not represent actual costs. *Third*, the SHARE study had approximately 37,000 and 1200 individuals responded to the survey in WAVE2 and WAVE3 for total 13 countries. This leads to a small sample size and a low statistical power for each country, especially for those who were severely limited in daily activities and who were terminally ill. Given this imprecision, one needs to be cautious in the interpretation of the estimates for informal care. *Lastly*, intangible costs, which value the loss of quality-adjusted life years for the affected patients, were not considered in this study.

### Implications for future economic evaluations

While COI analyses are useful for providing cost estimates for the impact of certain diseases, they cannot serve as the sole evidence for priority setting in terms of funding and resource allocation for prevention and treatment. Health economic evaluations for priority setting generally include both costs and outcomes in terms of survival and quality of life associated with specific interventions [24]. In 2018, NBHW assessed the cost-effectiveness of PCa screening on men aged 50–69 years in Sweden. Screening with the PSA test was considered to be cost-effective compared with no screening, but the assessment did not consider utilisation of new tests and complementary diagnostic tools and there was uncertainty for some key assumptions [29]. Due to these uncertainties, NBHW called for new economic evaluations of PCa screening using the new tests [29]. The resource and costs data from our study provide a reference for future economic evaluations to make informed decisions on whether to recommend a PCa screening program in Sweden. The values can also be used for economic evaluations for PCa therapies.

## Conclusion

The economic burden due to PCa was substantial and constitutes a major public health problem in Sweden. The two main cost drivers were direct medical care and informal care, which represent 58 and 32% of the total costs, respectively. The cost data in this study provide reference values for future economic evaluations for policy decisions to address the increasing public health problem of PCa.

## Supplementary information

**Additional file 1 Appendix A.** Combinations of ICD-10 codes and DRGs for health events identification in inpatient and outpatient care, Sweden. **Appendix B.** Drugs treating prostate cancer in Sweden. **Appendix C.** Palliative care. **Appendix D.** Informal care. **Appendix E** Productivity losses due to premature mortality. **Appendix F1.** Total prevalence, incidence and mortality of prostate cancer in Stockholm and Sweden. **Appendix F2.** Prevalence of prostate cancer by age group, Stockholm Region and Sweden, 2016.  **Appendix G.** Acknowledgement to SHARE. 

## Data Availability

The raw data used to estimate the resource utilisation of inpatient, outpatient and primary care, as well as the proportion of prescribed drug uses for prostate cancer patients during 2016 are available from the Stockholm PSA and Biopsy Register (SPBR). The aggregated prescribed and requisition drug costs data by substance level in 2016 from the Concise database that supported the data analyses of this study are available from the Swedish eHealth Agency. The data used to identify the proportion of prescribed drug uses of specific substances in 2012 for adult males are available from the Prescribed Drug Register (PDR). The data used to identify the proportion of requisition drug uses of specific substances prostate cancer patients are available from the Stockholm Electronic Patient Records Corpus (SEPR Corpus) Health Bank from Stockholm University. The data used to identify the resource utilisation of palliative care in the Stockholm region during 2016 are available from the Swedish Register of Palliative Care (SRPC). The data used to identify the resource utilisation of informal care of this study are available from the Survey of Health, Ageing and Retirement in Europe (SHARE) project. The data used to identify the workdays lost due to sick leave and early retirement of prostate cancer patients in the Stockholm region during 2016 are available from the Swedish Social Insurance Agency (FSK). Restrictions apply to the availability of these data, which were used under approval for the current study, and so are not publicly available. Anyone wishing to access the individual level data would need to apply for permission through an Ethical Review Board and from the primary data owners.

## Abbreviations

AS: Active Surveillance
ATC: Anatomical Therapeutic Chemical Classification System
COI: Cost-of-Illness
CPI: Consumer Price Index
DRE: Digital Rectal Examination
DRG: Diagnosis-Related Group
FSK: Swedish Social Insurance Agency
GP: General Practitioner
ICD-10: The International Classification of Diseases, 10th version
Mn: Million
MRI: Magnetic Resonance Imaging
NBHW: National Board of Health and Welfare
OR: Operation
PCa: Prostate Cancer
PDR: Prescribed Drug Register
PSA: Prostate-Specific Antigen
RP: Radical Prostatectomy
RT: Radiation Therapy
SCB: Statstics Sweden
SEPR Corpus: Stockholm Electronic Patient Records Corpus
SHARE: Survey of Health, Ageing and Retirement in Europe
SPBR: Stockholm PSA and Biopsy Register
SRPC: Swedish Register of Palliative Care
SRPL: Stockholm Region Price List
TLV: Dental and Pharmaceutical Benefits Agency
WW: Watching Waiting
