# Supplementary material for: The economic burden of prostate cancer – a Swedish prevalence-based register study
Source: BMC Health Serv Res. 2020 May 20;20:448. doi: 10.1186/s12913-020-05265-8 (PMC7238534; doi:10.1186/s12913-020-05265-8)
Supplement: Supplementary file 1 — Additional file 1 Appendix A. Combinations of ICD-10 codes and DRGs for health events identification in inpatient and outpatient care, Sweden. Appendix B. Drugs treating prostate cancer in Sweden. Appendix C. Palliative care. Appendix D. Informal care. Appendix E Productivity losses due to premature mortality. Appendix F1. Total prevalence, incidence and mortality of prostate cancer in Stockholm and Sweden. Appendix F2. Prevalence of prostate cancer by age group, Stockholm Region and Sweden, 2016. Appendix G. Acknowledgement to SHARE. [file 12913_2020_5265_MOESM1_ESM.docx]

**Appendix A. Combinations of ICD-10 codes and DRGs for health events identification in inpatient and outpatient care, Sweden**

| **ICD-10** | **Inpatient care - by DRG** | **Remarks** |
| --- | --- | --- |
| C61.9 | N01N Radical prostatectomy |  |
|  | N05N Transurethral resection of prostate |  |
|  | N10C Testes malignancy, operation (OR) procedures, with complications |  |
|  | N30C Reproductive system malignancy, OR procedures, with complications |  |
|  | N30E Reproductive system malignancy, OR procedures |  |
|  | N40C Reproductive system malignancy, other procedures, with complications |  |
|  | N40E Reproductive system malignancy, other procedures |  |
| N/A | N05N Transurethral resection of prostate (Non-primary) | For RT used at inpatient care, DRG codes N05N, R40C and R40E were applied irrespective of whether the primary diagnosis was PCa or not. |
|  | R40C Radiation therapy, with complications (Non-primary) |  |
|  | R40E Radiation therapy (Non-primary) |  |
| **ICD-10** | **Outpatient care - by DRG** | **Remarks** |
| C61.9 | N32O Reproductive system malignancy, OR procedures |  |
|  | N40O Reproductive system malignancy |  |
|  | N99X Team consultation for diseases of male genitalia |  |
|  | N99O Specialist consultation for diseases of male genitalia (Non-primary) |  |
| N/A | N75O Biopsy, male genitalia, including consultation | We used SPBR to identify the number of patients, the number of biopsies and the number of episodes related to prostate biopsies. The number of episodes included the biopsies conducted both at hospital outpatient care and at specialised outpatient clinics. The unit cost of N75O was used for calculating the total costs of episodes related to prostate biopsies. |
| Z51.0 | X11O Radiation therapy, resource-intensive (Non-primary) | For RP used at outpatient care, ICD-10 code Z51.0 (Radiotherapy) was used in combination with the related DRG codes X11O, X12O and X14O. |
|  | X12O Radiation therapy, including preparatory measures (Non-primary) |  |
|  | X14O Radiation therapy, less resource-intensive (Non-primary) |  |

ICD-10: The International Classification of Diseases, 10th version;

DRG: Diagnosis related group;

RT: Radiation therapy;

RP: Radical prostatectomy

**Appendix B. Drugs treating prostate cancer in Sweden**

**B1 Substance list for treating prostate cancer in Sweden, 2016 (1-5)**

| **ATC5** | **Substance English**  **(Swedish)** | **EMA approval** | **Other**  **indication** | **Mode of Administration** | **Main use** | **Estimated costs% - Prescribed, PCa^1^** | **Estimated costs% -**  **Requisition, PCa^1^** | **Estimated usage% -**  **Metastatic PCa^2^** | **Type of drug** |
| --- | --- | --- | --- | --- | --- | --- | --- | --- | --- |
| L01CD02 | [Docetaxel](https://www.cancer.gov/about-cancer/treatment/drugs/docetaxel)  ([Docetaxel](https://www.cancer.gov/about-cancer/treatment/drugs/docetaxel)) | 1995 | Yes | Intravenous infusion | Requisition | 7.1% | 28.6% | 100% | CT, Cytostatics |
| L01CD04 | [Cabazitaxel](https://www.cancer.gov/about-cancer/treatment/drugs/cabazitaxel)  (Kabazitaxel) | 2011 | No | Intravenous infusion | Requisition | N/A | 100.0% | 100% | CT, Cytostatics |
| L01DB07 | [Mitoxantrone](https://www.cancer.gov/about-cancer/treatment/drugs/mitoxantronehydrochloride)  (Mitoxantron) | 1987 | Yes | Intravenous infusion | Requisition | N/A | 100.0% | 0% | CT, Cytostatics |
| L02AA02 | Polyestradiol phosphate  (Polyöstradiolfosfat) | 1960 | Yes | Intramuscular injection | Prescribed | 96.8% | 28.6% | 0% | HT, Estrogen receptor agonists |
| L02AE01 | Buserelin  (Buserelin) | 1988 | Yes | Subcutaneous injection | Prescribed | 29.2% | N/A | 100% | HT, LHRH agonists |
| L02AE02 | [Leuprolin](https://www.cancer.gov/about-cancer/treatment/drugs/leuprolideacetate)  ([Leuprolin](https://www.cancer.gov/about-cancer/treatment/drugs/leuprolideacetate)) | 1987 | Yes | Subcutaneous injection | Prescribed | 95.2% | 100.0% | 33% | HT, LHRH agonists |
| L02AE03 | [Goserelin](https://www.cancer.gov/about-cancer/treatment/drugs/goserelinacetate)  ([Goserelin](https://www.cancer.gov/about-cancer/treatment/drugs/goserelinacetate)) | 1988 | Yes | Subcutaneous injection | Prescribed | 65.6% | 33.2% | 50% | HT, LHRH agonists |
| L02AE04 | Triptorelin  (Triptorelin) | 1996 | Yes | Subcutaneous injection | Prescribed | 46.7% | 35.1% | 50% | HT, LHRH agonists |
| L02BB01 | [Flutamide](https://www.cancer.gov/about-cancer/treatment/drugs/flutamide)  (Flutamid) | 1993 | Yes* | Oral | Prescribed | 100.0% | 100.0% | 50% | HT, Antiandrogens |
| L02BB03 | [Bicalutamide](https://www.cancer.gov/about-cancer/treatment/drugs/bicalutamide)  (Bikalutamid) | 1996 | No** | Oral | Prescribed | 99.8% | 100.0% | 20% | HT, Antiandrogens |
| L02BB04 | [Enzalutamide](https://www.cancer.gov/about-cancer/treatment/drugs/enzalutamide)  (Enzalutamid) | 2013 | No | Oral | Prescribed | 100.0% | 100.0% | 100% | HT, Antiandrogens |
| L02BX02 | [Degarelix](https://www.cancer.gov/about-cancer/treatment/drugs/degarelix)  ([Degarelix](https://www.cancer.gov/about-cancer/treatment/drugs/degarelix)) | 2009 | No | Subcutaneous injection | Requisition | 100.0% | 100.0% | 0% | HT, LHRH antagonists |
| L02BX03 | [Abiraterone](https://www.cancer.gov/about-cancer/treatment/drugs/abirateroneacetate)  (Abirateron) | 2011 | No | Oral | Prescribed | 100.0% | 100.0% | 100% | HT, Androgen synthesis inhibitors |

^1^ Estimates from this study;

^2^ Based on FASS (Farmaceutiska Specialiteter i Sverige) and expert opinion;

* Very little use for men who have had their testicles surgically removed;
** Also has indications not listed in the medication guide;
CT: Chemotherapy;
HT: Hormone therapy

**B2: Illustration of databases and steps to calculate drug costs due to prostate cancer**

PCa: Prostate Cancer;

TLV: The Dental and Pharmaceutical Benefits Agency;

PDR: Prescribed Drug Register;

SPBR: Stockholm PSA and Biopsy Register;

SEPR Corpus: Stockholm Electronic Patient Records Corpus

Several *prescribed drugs* had multiple indications, including Docetaxel, Polyestradiol phosphate, Buserelin, Leuprorelin, Goserelin and Triptorelin, which are also used to treat other indications for women and children younger than 10 years (1). To identify the proportion of drug uses by males at and above age 18 years, we used the Prescribed Drug Register, which contains information on the drug uses including details of the form, strength, dose, pack and brand, as well as age and sex (4). The drug uses by brand, age and sex were multiplied by the unit costs from The Dental and Pharmaceutical Benefits Agency (TLV) drug database (3). Additionally, Triptorelin was also used for chemical castration in other settings (1). We used the diagnosis and drug use information from SPBR to estimate the proportion of costs for Triptorelin used for PCa.

Docetaxel, Cabazitaxel, Mitoxantrone and Degarelix were categorised mainly as requisition drugs in Sweden (4). Docetaxel and Mitoxantrone were also used for treatment of other diseases (1). To estimate the costs due to PCa, data were extracted from the Stockholm Electronic Patient Records (SEPR) Corpus from Stockholm University (6). The proportion of usage due to PCa was calculated as the usage of a specific substance, form, strength and dose with a diagnosis of ICD-10 C61.9 divided by the total usage of this substance. The proportion of usage due to PCa was then multiplied by the substance costs from the Concise Database.

**Appendix C. Palliative care**

**C1: Illustration of selected questions from EQL (*partial*) (7)**

| 4. Date of death (year/month/day)_______________  5. Date (year/month/day) when the person was admitted to the unit where the death occurred (for home care, please state the date when home care was initiated)_______________  6. The place of death is best described as  □ Nursing home—permanent stay □ Nursing home—short-term stay □ Hospital ward (not hospice/palliative inpatient care) □ Hospice/palliative inpatient care □ Own home with support from specialised home-care team □ Own home with support from general home-care team □ Other, specify_______________  7. Disease/basic state that caused the death (more than one answer is possible):  □ Cancer □ Cardiovascular disease □ Respiratory disease □ Dementia □ Stroke □ Other neurological disease □ Diabetes □ State after fracture □ Multimorbidity □ Other, namely:_______________ |
| --- |

The question 6 listed the place where the diseased patient received palliative care. The selection “Hospital ward (not hospice/palliative inpatient care)” refers to hospital inpatient care.

**C2: Methods of calculation of palliative care costs due to prostate cancer**

The total costs of palliative care due to prostate cancer, denoted by C here, consists of two parts *C_n_* and *C_m_*_._ Mathematically,

*C = C_n_ + C_m_*

*C_n_* denoted the palliative costs from the linked records between SPRB and SRPC, *C_m_* represented the estimation of the potential palliative costs for those *not* reported to SRPC

*C_n_ = ∑ (D_ij *_ P_j_)*

where *i* = 1 to *n* and *n* indicated the number of patients with linked record; *j* = 1 to 5, where 1 = Hospice/palliative inpatient care, 2= Home support by daily contract of home service; 3= Home support with specialised palliative care; 4= Nursing home - permanent stay, 5 = Nursing home – Short-term stay. And

*C_m_ = (C_n_ /n) m*

where *m* represented the number of patients without palliative reported to SRPC. However, it is uncertain whether the proportion of different types of palliative care of those not reported cases would be the same as those reported to SRPC. *C_n_* was used for the base case. *C_m_* was estimated in a sensitivity analyses.

**Diseased due to PCa**

**386**

**Not reported**

**119 (30.8%)**

**Palliative care –**

**Others 102 (85.4%)**

- 155,703 x 102 = **15,8Mn SEK**

**Appendix D. Informal care**

**D1: Estimation of hours of informal care for PCa patients severely limited in daily activities was performed by adding the age-specific (age group) products of:**

1) Prevalence of prostate cancer in Stockholm in 2016, using data from the Stockholm PSA and Biopsy Register (SPBR);

2) Predicted probability of being severely limited in daily activities due to cancer: logistic regression after adjusting for age, sex, presence of cancer, presence of comorbidities and country of residence, using data from WAVE2 of SHARE;

3) Predicted probability of receiving informal care due to cancer: logistic regression after adjusting for age, sex, presence of cancer, presence of comorbidities and country of residence; two logistic regression were performed for i) informal care provided by someone inside the household and ii) informal care provided by someone outside the household, using data from WAVE2 of SHARE;

4) Predicted number of hours of informal care receive: linear regression adjusting for age, sex, presence of cancer, presence of comorbidities and country of residence; two linear regressions were performed for i) informal care provided by someone inside the household and ii) informal care provided by someone outside the household, using data from WAVE2 of SHARE; for how the hours were calculated for each individual, see Appendix D3;

5) Predicted probability of the time of the informal care is greater than zero: linear regression after adjusting for age, sex, presence of cancer, presence of comorbidities and country of residence, using data from WAVE2 of SHARE;

6) Predicted probability of care provider at working age: linear regression adjusting for age, sex, presence of cancer, presence of comorbidities and country of residence, using data from WAVE2 of SHARE. For details on the calculation of the probability of the care provider at working age, see Appendix D4.

**D2: Estimation of hours of informal care for PCa patients who were terminally ill was performed by adding the age-specific (age group) products of:**

1) Number of cancer death in Stockholm in 2016, using data from SPBR;

2) Predicted probability of obtaining informal care in the last year before death from cancer: logistic regression after adjusting for age, sex, cancer as cause of death and country of residence, using data from WAVE3 of SHARE;

3) Predicted number of total hours of informal care received due to cancer: linear regression after adjusting for age, sex, cancer as cause of death and country of residence, using data from WAVE3 of SHARE; for how the hours of informal care were calculated for each individual, see Appendix D5;

4) Predicted probability of care provider at working age: linear regression adjusting for age, sex, cancer as main cause of death and country of residence, using data from WAVE3 of SHARE; for details on the calculation of the probability of the care provider at working age, see Appendix D6.

According to SHARE, questions in WAVE3 were answered by proxy respondents including:

1) Husband or wife or partner; 2) Son or daughter; 3) Son- or daughter-in-law; 4) Son or daughter of husband, wife or partner; 5) Grandchild; 6) Sibling; 7) Other relative or 8) Other non-relative.

**D3: Calculations of hours of informal care for cancer patients limited in daily activities**

In WAVE2 of SHARE, each individual was asked about the frequency of informal care received and the corresponding hours under the specified care frequency. The frequency of informal care was categorised as “Almost daily”, “Almost weekly”, “Almost monthly” and “Almost yearly”. Respondents reported at most three persons who provided informal care. Total hours of informal care received in a year were not reported for each respondent.

In this study, we assumed 24 hours as the ceiling value for “Almost daily”; 168 (24*7) hours as the maximum value for “Almost weekly”; 720 (24*30) hours as the maximum value for “Almost monthly” and 8,760 (24*365) hours as the maximum value for “Almost yearly”. There were some outliers of the hours reported to WAVE2 of SHARE that were higher than the maximum hours of specific frequency. We believed that these respondents may have reported the total hours received rather than the estimated hours under the specified care frequency. We used the following table for the conversion of hours of each person provided informal care.

| **Frequency of informal care** | **Range of reported hours** | **Max hours based on the frequency** | **Reported hours**  **<= Max hours** | **Reported hours**  **> Max hours** |
| --- | --- | --- | --- | --- |
| Almost daily | 0 – 1800 | 0-24 (24*1) | reported hrs * 365 | reported hours |
| Almost weekly | 0 – 365 | 0-168 (24*7) | reported hrs * 52 | reported hours |
| Almost monthly | 0 – 200 | 0-720 (24*30) | reported hrs * 12 | N/A |
| Almost yearly | 0 – 600 | 0-8760 (24*365) | reported hrs * 1 | N/A |

For each respondent, we added the converted annual hours received from each care givers. The table below shows examples of two respondents who received informal care.

| **Respondent** | **Care giver** | **Frequency of informal care** | **Reported hours** | **Converted annual hours** | **Total annual hours received** |
| --- | --- | --- | --- | --- | --- |
| 1 | 1 | Almost daily | 1,600 | 1,600 | 4,760  (1600 + 2,920 + 240) |
|  | 2 | Almost daily | 8 | 2,920 (8*365) |  |
|  | 3 | Almost monthly | 20 | 240 (20*12) |  |
| *2* | 1 | Almost yearly | 100 | 100 (100*1) | 100 |
|  | 2 | N/A |  |  |  |
|  | 3 | N/A |  |  |  |

**D4 Calculations of probability of care giver was at working age**

Following the same assumption from the European study (8) and a Swedish report (9), for respondents or diseased person aged less than 65 years, we assumed that the help from their spouse/ex-spouse, friends, children (including son/daughter in-law), grandchildren, niece or nephew, siblings cousins, neighbours and unpaid volunteer were aged less than 65. For respondents or diseased person at aged 65 years or over, informal care provided by children (including son/daughter in-law), grandchildren, niece/nephew and unpaid volunteer were assumed to be aged less than 65. Using these assumptions, the probability of receiving informal care from someone at working age was calculated by the number of care givers at working age divided by the total number of persons provided informal care to the respondent. See examples from the table below.

| **Respondent** | **Age** | **Care giver** | **Working age** | **Total number of care giver** | **Calculated probability of receiving informal care from someone at working age** |
| --- | --- | --- | --- | --- | --- |
| 1 | 70 | Son-in-law | Yes (1) | 3 | 1/3= 33.3% |
|  |  | Sibling | No (0) |  |  |
|  |  | Neighbour | No (0) |  |  |
| *2* | 62 | Husband | Yes(1) | 1 | 1/1=100% |
|  |  | N/A | N/A |  |  |
|  |  | N/A | N/A |  |  |

**D5: Calculations of hours of informal care for cancer patients who were terminally ill**

In WAVE3 of SHARE, each respondent reported the total time that they have provided unpaid care to someone died in the last year. The frequency of informal care provided was categorised as “less than one month”, “between one and three months”, “between three and six months”, “between six months and a full year” and “a full year”. In this study, we used the following assumptions for converting the hours received by the diseased person.

| **Total duration of informal care** | **Number of days assumed for the specified duration** | **Conversion of total hours provided to the diseased person** |
| --- | --- | --- |
| < 1 month | 15 | reported hours * 15 |
| 1 – 3 months | 60 | reported hours * 60 |
| 3 – 6 months | 135 | reported hours * 135 |
| 6 – 12 months | 270 | reported hours * 270 |
| 1 full year | 360 | reported hours * 360 |

**D6 Calculations of probability of care giver was at working age for cancer patients who were terminally ill**

Following the same assumption from the European study (8) and a Swedish report (9), for respondents or diseased person aged less than 65 years, we assumed that the help from their spouse/ex-spouse, friends, children (including son/daughter in-law), grandchildren, niece or nephew, siblings cousins, neighbours and unpaid volunteer were aged less than 65 years. For respondents or diseased person aged 65 years or over, informal care provided by children (including son/daughter in-law), grandchildren, niece/nephew and unpaid volunteer were assumed to be aged less than 65. Using these assumptions, the probability of receiving informal care from someone at working age was calculated by the number of care givers at working age divided by the total number of persons provided informal care to the respondent. Examples were showed in the table below.

| **Diseased person** | **Age** | **Care giver** | **Working age** | **Total number of care giver** | **Probability of informal care provided by someone at working age** |
| --- | --- | --- | --- | --- | --- |
| 1 | 70 | Partner | No (0) | 3 | 2/3= 66.7% |
|  |  | Grandchildren | Yes (1) |  |  |
|  |  | Unpaid volunteer | Yes (1) |  |  |
| *2* | 62 | Friend | Yes(1) | 1 | 1/1=100% |
|  |  | N/A | N/A |  |  |
|  |  | N/A | N/A |  |  |

**Appendix E Productivity losses due to premature mortality**

Mathematically, let the age at prostate cancer death be *i*, with number of deaths $d_{i}$, assuming deaths are in the middle of each one-year age group, and the background population mortality $\mu_{0}(s)$ at age *s* from vital statistics, with discount rate $\delta=0.03$. Then the discounted potentially productive years of life lost (PPYLL), adjusted for survival, is

$$\text{PPYLL}=\sum_{i=0}^{64} d_{i}\int_{i+0.5}^{65} \frac{\exp\left( -\int_{i+0.5}^{t} \mu_{0}\left( s \right)ds \right)}{\left( 1+\delta\right)^{t-\left( i+0.5 \right)}}dt=\sum_{i=0}^{64} d_{i}\int_{i+0.5}^{65} \exp\left( -\int_{i+0.5}^{t} \left( \mu_{0}\left( s \right)+log\left( 1+\delta\right) \right)ds \right)dt$$

**Appendix F1: Total prevalence, incidence and mortality of prostate cancer in Stockholm and Sweden (10)**

**Appendix F2: Prevalence of prostate cancer by age group, Stockholm Region and Sweden, 2016 (10)**

Proportion per 100,000

**Appendix G. Acknowledgement to SHARE**

This paper uses data from SHARE Waves 2 and 3 (DOIs: [10.6103/SHARE.w2.700](http://dx.doi.org/10.6103/SHARE.w2.700), [10.6103/SHARE.w3.700](http://dx.doi.org/10.6103/SHARE.w3.700)), see Börsch-Supan et al. (2013, as cited in the main reference list) for methodological details.

The SHARE data collection has been funded by the European Commission through FP5 (QLK6-CT-2001-00360), FP6 (SHARE-I3: RII-CT-2006-062193, COMPARE: CIT5-CT-2005-028857, SHARELIFE: CIT4-CT-2006-028812), FP7 (SHARE-PREP: GA N°211909, SHARE-LEAP: GA N°227822, SHARE M4: GA N°261982) and [Horizon 2020](https://cordis.europa.eu/guidance/archive_en.html) (SHARE-DEV3: GA N°676536, SERISS: GA N°654221) and by DG Employment, Social Affairs & Inclusion. Additional funding from the German Ministry of Education and Research, the Max Planck Society for the Advancement of Science, the U.S. National Institute on Aging (U01_AG09740-13S2, P01_AG005842, P01_AG08291, P30_AG12815, R21_AG025169, Y1-AG-4553-01, IAG_BSR06-11, OGHA_04-064, HHSN271201300071C) and from various national funding sources is gratefully acknowledged*(see* [*www.share-project.org*](http://www.share-project.org/)*).*

**REFERENCES**

1. FASS [Internet]. Farmaceutiska Specialiteter i Sverige. 2018 [cited 2019 Mar 21]. Available from: <https://www.fass.se/LIF/startpage>.

2. National cancer institute. Drugs approved for prostate cancer: National cancer institute; 2018 [updated 2018 Jun 13; cited 2018 Jul 1]. Available from: <https://www.cancer.gov/about-cancer/treatment/drugs/prostate>.

3. Läkemedel [Internet]. Tandvårds- och läkemedelsförmånsverket. 2018 [cited 2018 Jul 1]. Available from: <https://www.tlv.se/beslut/sok-i-databasen.html>.

4. Vårdanalys. Cancerläkemedel – ett kunskapsunderlag om införande, användning och uppföljning. The Swedish Agency for Health and Care Services Analysis; 2017.

5. Medicines [Internet]. European Medicines Agency. 2018 [cited 2019 Apr 10]. Available from: <https://www.ema.europa.eu/en/medicines>.

6. Dalianis H, Hassel M, Velupillai S. The Stockholm EPR Corpus Characteristics and Some Initial Findings. In: Proceedings of ISHIMR 2009, 14th International Symposium for Health Information Management Research, Kalmar. 2009.

7. Martinsson L, Heedman PA, Lundstrom S, Axelsson B. Improved data validity in the Swedish Register of Palliative Care. PLoS One. 2017;12(10):e0186804.

8. Luengo-Fernandez R, Leal J, Gray A, Sullivan R. Economic burden of cancer across the European Union: a population-based cost analysis. Lancet Oncol. 2013;14(12):1165-74.

9. Lundqvist A, Andersson E, Carlsson K. Kostnader för cancer i Sverige idag och år 2040. IHE; 2016. Report No.: 1651-7598.

10. Cancer statistics for the Nordic countries [Internet]. Association of the Nordic Cancer Registries. 2017 [cited 2019 Mar 13]. Available from: <http://www-dep.iarc.fr/NORDCAN/English/frame.asp>.
